# Accessing the growth of heat‐resistant mold ascospores in potato dextrose agar, isolated from a processed fruit jam—Dependence of temperature, pH, and sugar concentration

**DOI:** 10.1002/fsn3.4486

**Published:** 2024-10-07

**Authors:** Mahabub Alam, Animesh Sarkar, A. S. M. Sayem, Rahul Biswas, M. M. Mahdi Hasan

**Affiliations:** ^1^ Department of Food Engineering and Tea Technology Shahjalal University of Science and Technology Sylhet Bangladesh

**Keywords:** heat‐resistant molds, molecular identification, processed fruits, processing condition, time to detectable growth

## Abstract

Heat‐resistant molds (HRM) pose a significant threat to food industries due to their ability to survive in high temperatures (pasteurization range) and grow in a variety of environments. To understand how inhibiting variables affect HRM growth, this study evaluated the impact of high sugar concentration (50–60°Brix), low pH (3.5–4.5), and temperature (5–15) on the time (tv) to develop detectable colonies (colony diameter = 2 mm) of three HRM strains (*Byssochlamys nivea*, *Byssochlamys fulva*, *Neosartorya fischeri*) isolated from mixed fruit products. The study found that all HRM strains had relatively short periods of time to develop detectable colonies at 50°Brix, while no visible growth was observed at 60°Brix. *Byssochlamys nivea* was found to be the most susceptible to low temperatures, requiring up to 43 days to develop detectable colonies at 10°C and unable to grow at colder temperatures. The tv varied from 6 to 27 days based on pH and isolates. HRM's resistance to high sugar concentrations and ability to grow in cold environments pose a threat to the microbiological safety of fruit products. The evaluated data were fitted to several statistical distribution models to support further research on the quantitative microbiological risk in processed fruit products. This study provides valuable insights into how inhibiting variables affect HRM growth and can inform efforts to minimize their impact on fruit‐based product quality and safety.

## Graphical abstract

Heat‐resistant molds (HRMs) pose a significant threat to processed fruits. Effect of processing conditions on time to detectable growth (tv) of HRMs was determined. Risk software was used to fit probability distribution functions to the cumulative tvs. These data may be used to evaluate and predict microbiological risk to prevent spoilage.

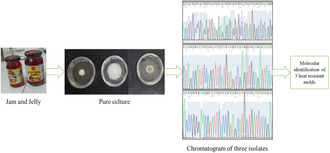

## 1 INTRODUCTION

Globally, fruit processing is a widespread practice, transforming fruits into products such as jam, jelly, marmalade, and fruit juice. This process boosts economies by generating significant economic activity and ensures access to fruits even during off‐season periods (Shinwari & Rao, 2018). Despite the significant economic benefits associated with the production of these products, they are particularly susceptible to microbial spoilage, mainly from heat‐resistant molds (HRM). HRM have been identified as a major challenge to the fruit‐based products manufacturing industry due to their thermal resilience and ability to thrive in diverse environments (Santos et al., 2020). Therefore, it is crucial to understand how inhibiting variables impact HRM development to prevent spoilage during the product's storage period.

HRM are known as specific spoilage microbes (SMOs) of thermally processed fruit commodities worldwide. They are classified as SMOs due to their frequent occurrence in fruit products, ability to resist pasteurization procedures typically used in fruit processing, and ability to overcome the barriers implemented by fruit processors (Dos Santos et al., 2018). Various preservation techniques are implemented to combat HRM, including reducing water activity (often achieved by adding sugar), lowering pH levels through organic acid addition, limiting oxygen levels, incorporating antimicrobial agents, and storing the foodstuff in refrigerated environments, among others (Berni et al., 2017). In most cases, strong heat treatments and barriers like those described above are sufficient to completely block most spoilage bacteria. However, these treatments and circumstances may not be enough to stop HRM from growing. If ascospores remain on the goods after pasteurization and are exposed to suitable circumstances, they can grow and develop. Their proliferation is followed by the appearance of apparent mycelium on the food's surface, as well as organoleptic deterioration, which leads to customer refusal and financial losses. Mycotoxins have been found in a variety of HRM species (Frąc et al., 2015). Mycotoxins, including aflatoxins, ochratoxin A and patulin, are significant contaminants in thermally processed fruit commodities. These toxins pose substantial health risks due to their carcinogenic, nephrotoxic, hepatotoxic and neurotoxic effects, among others (Singh & Mehta, 2020). For instance, aflatoxins are potent carcinogens associated with liver cancer, while ochratoxin A is known for its nephrotoxic effects and potential links to kidney disease (Kortei et al., 2021). Patulin, commonly found in apple products, has been implicated in gastrointestinal disorders and immunotoxicity (Przybylska et al., 2021).

Many experiments have been performed on the effect of environmental variables on HRM development by estimating fungal growth rates and lag periods, which are frequently of high inoculum (Santos et al., 2018). However, these impacts on HRM time to visible growth, or the time it takes to expand to a stage that customers can perceive, have received less attention. Nonetheless, fruit processors are interested in this characteristic since it may be used as a technique to predict the mold‐free shelf‐life (Dantigny, 2016). Only one or a few spores are sufficient to contaminate goods via HRM (Dos Santos et al., 2018). As a result, in research aimed at determining mold‐free shelf life, biological variability of individual ascospore must be considered as a stochastic process.

Some authors have focused on the biological diversity of single mold spores from the same community, highlighting the need of conducting individual spore development kinetics investigations (Dagnas et al., 2017; Garcia et al., 2010). However, there is no information on how long it takes for individual ascospores to become visible in Bangladeshi industrially processed fruit items. This study provides new insights into the development of heat‐resistant molds (HRM) isolated from fruit items and how inhibiting variables affect their growth. Specifically, the study evaluates the highest sugar concentration, lowest pH, and temperature for the development of three HRM and calculates the time to develop a detectable colony (tv) to evaluate the biological heterogeneity of individual ascospores within the same species. The findings of the study suggest that HRM poses a significant threat to fruit‐based product manufacturers due to their resistance to elevated sugar concentrations and their ability to grow in cold environments. The study also highlights the importance of understanding how inhibiting variables affect HRM development to minimize product deterioration during storage periods. The data collected in this study can inform efforts to minimize the impact of HRM on fruit‐based product quality and safety, and further research can be conducted to quantitatively assess the risk of microbial deterioration in pasteurized fruit products. The goal of this research was to isolate and characterize the heat‐resistant molds from processed fruit products (mixed fruit jam) as well as to evaluate the effect of processing conditions (°Brix, temperature, pH) on time to detectable growth (tv) of individual ascospores in acidified potato dextrose agar media.

## 2 MATERIALS AND METHODS

### 2.1 Sampling

The mixed fruit jam was purchased from locally processed (pasteurization range) industry. The initial pH and °Brix of the jam were 4.37 and 54.25, respectively. Until the analysis was done, it was kept at −20°C.

### 2.2 Isolation and collection of HRM

According to Dos Santos et al. (2018) technique, heat‐resistant molds were isolated from mixed fruit jam. The presence of heat‐resistant fungal ascospores was examined in 10 g of each sample. First, 15 mL of sterile, distilled water was used to dilute the samples that were placed in stomacher bags. The diluted samples were subsequently homogenized in a stomacher for a duration of 2 min and then heat‐sealed. Following sealing, the bags underwent a 30‐min heat treatment at 80°C in a water bath. Subsequently, under aseptic conditions, the samples were promptly transferred to sterilized Schott bottles filled with 250 mL of molten double‐strength Malt Extract Agar (MEA) tempered to 55°C. Prior to use, the Schott bottles were sterilized. The agar medium was enriched with 200 mg/L of chloramphenicol to inhibit bacterial growth. Following the heat treatment, the samples were thoroughly mixed with agar and evenly spread onto Petri plates. Each plate was then tightly sealed with parafilm and placed inside a plastic bag to prevent dehydration during the incubation period. The plates were subsequently placed in an incubator set at 30°C for up to 30 days, with visual inspections conducted every 7 days to monitor microbial growth. Using a sterile inoculation loop, all colonies isolated from the examined specimens were selected, streaked onto MEA plates, and cultured for 7 days at 30°C. This was carried out repeatedly until pure cultures (isolates), or the formation of colonies with the same macroscopic features such as colony size, color, and appearance, were obtained.

### 2.3 Molecular identification of fungal ascospores

**FIGURE 1:**
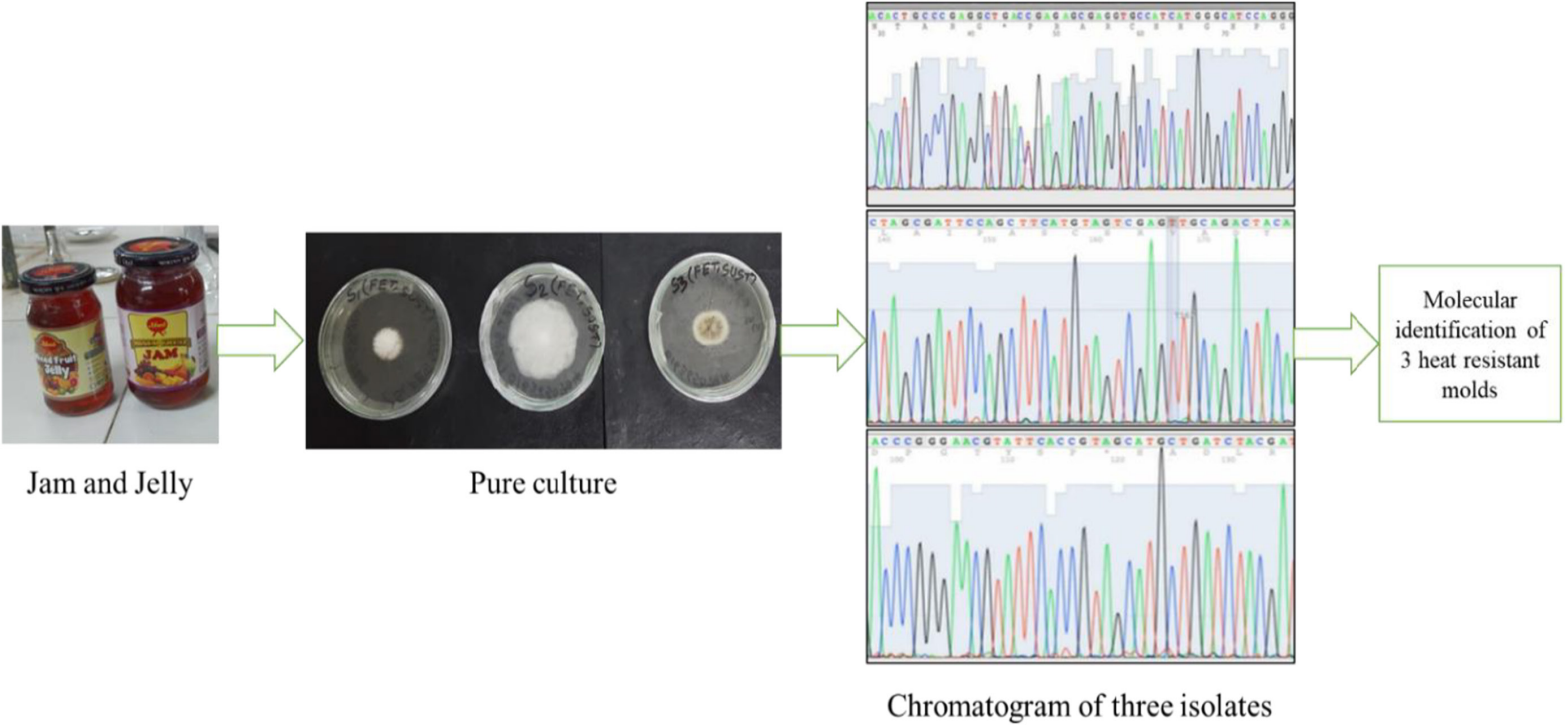
Isolation and characterization of heat‐resistant molds from processed mixed fruit jam.

The HRM were identified at the species level via gene sequencing (Figure 1). DNA extraction, DNA amplification, and DNA sequencing were the three steps in the sequencing process. First, the isolates were cultured in 12‐well microplates for 14 days at 25°C in 3 mL of potato dextrose broth (PDB). After centrifuging the resultant mycelial mats for 5 min at 8000*g* and 4°C, they were collected in Eppendorf tubes. The resultant pellets were then lyophilized for 20 h at −80°C to create a fine powder. Then, using the Wizard Genomic DNA Purification Kit (Promega, Madison, USA) in accordance with the manufacturer's instructions, the genomic DNA was recovered. After DNA extraction, the universal primers ITS4 (5′‐TCCTCCGCTTATTGATATGC‐3′) and ITS5 (5′‐GGAAGTAAAAGTCGT AACAAGC‐3′) were used to amplify the DNA. By adding 2 μL of genomic DNA to 23 μL of the reaction mixture, the PCR amplification procedures were carried out. A FlexCycler PCR thermocycler was used for the amplification, with the following settings: initial denaturation at 94°C for 10 min; 35 cycles of denaturation at 94°C for 60 s; annealing at 55°C for 60 s; elongation at 72°C for 60 s; and final chain elongation at 72°C for 10 min. Sanger sequencing was used by LGC Genomics GmbH (Berlin, Germany) to finally ascertain the sequences. Then, using BioEdit's (BioEdit 7, USA) sequence alignment editor, consensus sequences were produced. The National Center for Biotechnology Information's (USA) BLAST (Basic Local Alignment Search Tool) tool was then used to blast the consensus sequences. This tool can be accessed online at http://blast.ncbi.nlm.nih.gov. In order to determine sequence similarity, the BLAST tool compares the consensus sequences to those in its database.

### 2.4 Suspensions of ascospores formulation

To guarantee the production of ascospores, mold strains were cultured using suitable agar media for 30 days at 30°C. After soaking each Petri dish with sterile 0.1% Tween 80 (8 mL), filtering and centrifuging was conducted three times at 4°C for 15–20 min to produce ascospore suspension. After that, distilled water (10 mL) was added to make the final solution. After a 10‐min heating shock at 80°C, the concentration of ascospore suspensions was measured by serial dilutions method. The plates were enumerated 1 week after incubation at 30°C. The final suspensions were standardized based on the results of the following test, stored at 2–3°C, and utilized within a week of harvesting.

### 2.5 Effect of processing conditions on time to detectable growth of individual ascospores

**FIGURE 2:**
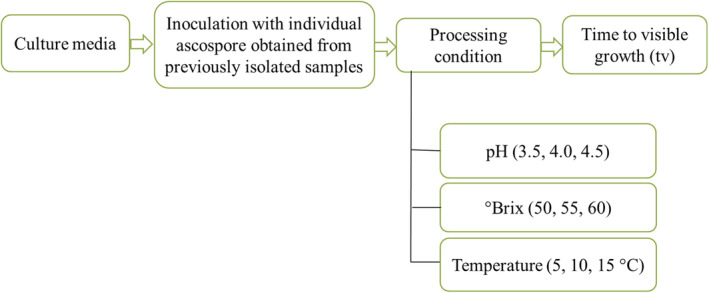
Schematic representation of the processing condition on time to detectable growth of individual ascospore.

Observations were made on the impact of various processing conditions, including °Brix, temperature, and pH, on the time required for individual ascospores to reach detectable growth (defined as a colony diameter of 2 mm) over a 45‐day period (Figure 2).

#### 2.5.1 Impact of °Brix on the time it takes for visible growth

#### 2.5.2 Impact of temperature on the time it takes for visible growth

For the temperature experiments, each HRM was suspended in a sterile buffer solution made by dissolving 70 g/L potassium dihydrogen phosphate in 1 L distilled water acidified to pH 3.5 using phosphoric acid. After being heat activated at 80°C for 10–15 min, aliquots of 100 μL of calibrated suspensions were distributed in triplicate on acidified PDA plate. The plates were sealed by using parafilm and subsequently incubated at 5, 10, and 15°C after inoculation. For up to 45 days, the plates were examined for apparent development (colony diameter 2 mm). Data on growth or no growth, time to visible growth as well as quantity of visible colonies was also evaluated. The experiment was repeated twice.

#### 2.5.3 Effect of pH on the time requires to visible growth

The pH of the media was adjusted to 3.5–4.5 using citric acid. Before inoculating into the media, the ascospore suspensions were adjusted to 100–105 spores using serial dilutions. For up to 45 days, the plates were examined for apparent development (colony diameter = 2 mm). Data on growth and no growth, as well as the time it took for apparent growth and the quantity of apparent colonies were also evaluated.

### 2.6 Statistical analysis

Statistical distributions were fitted to the data (number of observed colonies vs. time) for each processing condition by using @Risk 8.2 software. The probability distributions provided in @Risk were visually examined and graded using the Bayesian Information Criterion (BIC). The most accurate estimates for key parameters, including the mean, 95% confidence interval (CI), and standard deviation, were obtained for the distributions that had the best fits. By comparing the 95% confidence intervals for the tvs, significant differences (at α = 0.05) between them were identified.

## 3 RESULTS

Three strains of heat‐resistant molds such as *Byssochlamys fulva*, *Byssochlamys nivea* and *Neosartorya fischeri* were isolated and identified from the highly processed food products (mixed fruits Jam) of Bangladeshi food industry.

Based on information from the literature, the three °Brix values (50, 55, and 60) were chosen. However, visible mycelia were seen in our study between 4 and 30 days at 30°C, depending on the isolate and sugar concentration. The least susceptible to high sugar concentrations was *B. nivea*, which was unable to thrive at levels above 55°Brix. The significantly most tolerant isolates at 50°Brix levels were *N. fischeri*. All the HRM showed very short tvs (4–8 days) at the lowest sugar content of 50°Brix. No observable growth was found over a period of 45 days at 60°Brix.

Temperature effects ranging from 5 to 15°C were evaluated on the time to detectable growth of three isolates. None of the isolates were able to grow at 5°C over a period of 45 days. They started to grow at 10 to 15°C within 8–26 days. The most cold‐sensitive species was *B. nivea* since it could not germinate and produce visible growth at low temperatures and only showed visible colonies at 10°C after up to 43 days of incubation. It also exhibited significantly longer tv compared to other two isolates at 15°C. However, *N. fischeri* showed obvious outgrowth at 10°C immediately after 8–12 days, whereas *B. fulva* and *B. nivea* did so after 8–22 days and 17–35 days, respectively.

On PDA media, the three HRM strains' minimal growth pH (acidity tolerance) was assessed. Depending on the pH and isolates, the tv varied anywhere from 6 to 27 days. Overall, all isolates had significantly longer tv (*p* < .05) when the acidity increased from 4.5 to 3.5.

The findings of this investigation demonstrate the extreme variability of ascospores tv from the same isolates. Probability distributions expressing uncertainties and/or variabilities would thus be a better fit for this parameter's description. This study used the assumption that each visible colony found after a specified period (45 days) had its origins in a single ascospore. Thus, the approximately 100 tv values (from about 100 ascospores) for each isolate under each experimental condition were acquired. These tv's were adjusted in @Risk to suit statistical distributions. The most appropriate distributions were found (see Tables 1, 2, and 3). Most of the data on the impact of °Brix were better expressed by exponential curves defined at the estimated parameter “Risk shift,” that is, a shift value from 0. These curves were also characterized by a single scale parameter. Most likely values from exponential curves were clustered in the bottom border, which in this study indicates the shortest tvs seen for the most of the ascospores in an isolate. Additionally, depending on the quality of fit, other types of distributions were used in some circumstances (Table 1). For example, normal distributions provided the best fit for the *B. fulva* tv data at 50°Brix. A wider biological diversity among ascospores from the same population would be expected under these circumstances given that these variances in distributions were also connected with the growth/no growth zones in which larger tv ranges were found.

**TABLE 1:** Effect of Brix (%) on time to detectable growth of individual ascospores.

HRM	Brix (%)	a_w_	Distribution	*t*_v_ (days)	5th percentile	95th percentile
*Byssochlamys fulva*	50	0.903 ± 0.005	RiskNormal (8.7667,3.1535)	9 ± 3.15	4	14
	55	0.895 ± 0.006	RiskExpon (2.8172; RiskShift (8.6705))	11 ± 2.69	9	19
	60	0.868 ± 0.008	No growth	‐	‐	‐
*Byssochlamys nivea*	50	0.903 ± 0.005	RiskExpon (2.2739; RiskShift (6.9745))	11 ± 3.15	8	15
	55	0.895 ± 0.006	RiskExpon (5.9711; RiskShift (13.8289))	26 ± 2.95	18	30
	60	0.868 ± 0.008	No growth	‐	‐	‐
*Neosartorya fischeri*	50	0.903 ± 0.005	RiskExpon (1.8219; RiskShift (2.5473))	5 ± 1.62	4	15
	55	0.895 ± 0.006	RiskExpon (2.9136; RiskShift (8.7432))	14 ± 3.49	9	20
	60	0.868 ± 0.008	No growth	‐	‐	‐

**TABLE 2:** Effect of temperature (°C) on time to detectable growth of individual ascospores.

HRM	Temperature (°C)	Distribution	*t*_v_ (days)	5th percentile	95th percentile
*Byssochlamys fulva*	5	No growth	‐	‐	‐
	10	RiskLognorm (5.9726; 3.2176; RiskShift (24.1272))	30.1 ± 3.92	26	40
	15	RiskExpon (3.9375; RiskShift (6.8671))	10.2 ± 2.72	8	22
*Byssochlamys nivea*	5	No growth	‐	‐	‐
	10	RiskLogistic (39.9167; 3.9132)	40.4 ± 3.65	29	43
	15	RiskNormal (26.6376; 3.7437)	26.6 ± 3.74	17	35
*Neosartorya fischeri*	5	No growth	‐	‐	‐
	10	RiskExpon (2.95; RiskShift (18.7576))	22.4 ± 4.49	19	30
	15	RiskExpon (1.7090; RiskShift (6.9894))	10.15 ± 1.17	8	12

**TABLE 3:** Effect of pH on time to detectable growth of individual ascospores.

HRM	pH	Distribution	*t*_v_ (days)	5th percentile	95th percentile
*Byssochlamys fulva*	3.5	RiskWeibull(1.7135; 8.4612; RiskShift (10.5247))	17 ± 2.19	15	28
	4.0	RiskPareto (4.6733; 5)	9 ± 2.92	7	11
	4.5	RiskExpon (0.5671; RiskShift (6.11305))	7 ± 1.72	6	10
*Byssochlamys nivea*	3.5	RiskWeibull (1.7285; 8.4912; RiskShift (11.6747))	18 ± 2.73	14	27
	4.0	RiskLognorm (10.741; 3.6301; RiskShift (2.6221))	12 ± 2.51	9	20
	4.5	RiskExpon (0.5661; RiskShift (5.79605))	6 ± 1.93	6	10
*Neosartorya fischeri*	3.5	RiskExpon (3.6838; RiskShift (10.7928))	19 ± 1.86	13	25
	4.0	RiskExpon (0.86791; RiskShift (8.69827))	10 ± 2.49	9	13
	4.5	RiskExpon (2.5276; RiskShift (4.3855))	8 ± 2.17	6	15

The distribution and spread of individual tv appear to be more significantly impacted by a drop in temperature in the investigated range (5–10°C) than by a rise in sugar content (°Brix) in the evaluated range (50–60°Brix). Since most of the distributions were found to be exponential, shorter timeframes were the pattern for most tv values. The curves reported for the *Byssochlamys* isolates, on the other hand, were distinguished by broader tv ranges and were described by logistic, lognormal, and normal distributions (Table 2). While logistic curves and normal curves are similar, they differ in that they produce the most probable values in extreme circumstances (tails).

Nonetheless, four different types of distribution models namely, weibull, pareto, exponential, and lognormal were observed for different pH and isolates (Table 3). A decrease in pH in the studied range (3.5–4) seems to have less of an influence on the distribution and spread of individual tv than a fall in temperature or a rise in sugar content. Additionally, the individual ascospore tv's of *B. fulva*, which ranged from 7 to 11 days at pH 4, were expressed by Pareto distributions. It was distinguished by a shape and scale parameter (α, β): (4.67, 5). This distribution resembles an exponential distribution in that its density decreases from its mode in α at a rate β.

## 4 DISCUSSION

The preservation of pasteurized fruit products primarily relies on their high acidity and the application of heat during processing (Cheng et al., 2020). While this combination effectively eliminates and inhibits the growth of most spoilage microorganisms and pathogens, it may not completely eradicate spores of certain microorganisms (Dos Santos et al., 2018). Mixed fruit jam was examined to determine the frequency and variability of HRM contamination levels during manufacturing. Even though this study only detected a small number of HRM (*Byssochlamys* sp. and *Neosartorya* sp.), most are extremely important economically since they contribute to the spoiling of pasteurized high‐acid fruit items.

Fungi just needs a few basic nutrients to develop. After the ascospores are subjected to outside trigger like heat, chemicals, or high pressure, dormancy is typically broken (Wyatt et al., 2013). The breakdown of suitable solutes, a reduction in the cytoplasm's viscosity, and disintegration of the thick cell wall, which enable absorption of nutrients by the activated ascospores and the start of the germination process, are the hallmarks of the shift from an inactive to an active metabolic function. This includes the tube's development and extension, accompanied by hyphal outgrowth and branching (Burgain et al., 2013; Gougouli & Koutsoumanis, 2013). The time variable (tv) is frequently described as the point at when the mycelium diameter equals 2–3 mm, which refers to the lag time and the start of the linear growth (Gougouli et al., 2011). Consequently, there is a growing trend in studies aiming to predict fungal decay to replace the lag time for tv.

The primary component affecting fungal growth and germination is water activity (aw). Therefore, most studies that have been conducted on the development of HRM have evaluated this parameter (Berni et al., 2017; Panagou et al., 2010). The impact of sugar concentrations (°Brix) is used consistently in the fruit processing sector, even though aw is a valuable measure for food microbiologists. Notwithstanding this, little information about the impact of °Brix on the growth of HRM is presently available. *Neosartorya* strains' sugar concentration limiting parameters on fruit‐based media were recently identified by Berni et al. (2017). According to the authors, depending on the species, *Neosartorya* spp. might withstand sugar concentrations between 49 and 56°Brix. These values were slightly lower than what we saw in our investigation (55–60°Brix). *N. hiratsukae* was only able to grow in fruit‐based medium at Brix levels below 50, which is in contradiction to our findings. This could be due to variations in the media and strains used. Beuchat and Toledo (1977) examined how *B. nivea* ascospores behaved in fruit products enriched with sucrose (20%–60% soluble solids). According to the authors, *B. nivea* was unable to germinate in most of processed fruits having 60% total soluble solids, but after 3–13 days at 30°C and 4–34 days at 21°C, identifiable mycelia were found on fruit products having 40% sucrose (Beuchat & Toledo, 1977).

As thermotolerant microbes, HRM can thrive at temperatures as high as 45–50°C and as low as 20°C. *B. nivea* was suppressed in a variety of fruit products when incubated at or below 7°C, which is consistent with our findings. Most HRM have not been found to develop at 5°C, when chilling conditions are present. After approximately 20 days, three of our isolates were able to expand at 10°C. Some variables, such as substrate concentration, heat, and inoculum size, may have a significant impact on tv. At lower °Brix levels, *Neosartorya* strains had shorter detection times for apparent mycelium, according to Berni et al. (2017). For instance, *N. fischeri* showed apparent development after 4–15 days at 50°Brix. However, it has been shown in several research that when HRM are inoculated on separate (different kinds of) fruit matrices with the same aw‐value, identical growth characteristics are produced (Berni et al., 2017; Zimmermann et al., 2011). According to Beuchat and Toledo (1977) research, when *B. nivea* ascospores were inoculated in various fruit juices and nectars with equal aw values, different growth times were found. *B. nivea*, for instance, was able to generate visible mycelium after 17 days at 21°C when it was inoculated in apple juice (aw = 0.92 and 40% sucrose), but it only required four days in grape juice for the first mycelium to develop. In contrast, when the °Brix was as low as 48.5 (aw = 0.94), cranberry juice maintained at 21°C did not facilitate the growth of HRM (Beuchat & Toledo, 1977). Differences in composition may explain why the maximum °Brix values for HRM growth in fruit‐based media are lower compared to our findings. Fruits include organic acids and phenolic substances that may significantly affect how HRM develop (Amaeze, 2013; Panagou et al., 2010). The spread (interval) of each individual tv is defined by their inherent biological diversity and cannot be decreased by raising the size of experiments. As the circumstances got more unfavorable, we noticed larger spreads of the tvs of individual ascospores, which suggests that stress‐induced fungal growth is more variable. Although information on the biological variability of ascospores is currently lacking, heat‐sensitive spores have been known to exhibit this diversity (Dagnas et al., 2017; Garcia et al., 2010; Gougouli & Koutsoumanis, 2013). According to Dagnas et al. (2017), stressful situations cause a mismatch between individual and population lag times. They measured the inhibitory impact of aw and storage temperature on the lag durations of single spores of molds collected from spoiled bread items. Similar to this, Samapundo et al. (2007) examined the impact of aw and temperature on the distinct lag periods of molds linked to the spoiling of maize and discovered more variation (spread) on growth parameters under stressed circumstances. But it's also probable that uncertainty related to potential changes within replicates had a role in the spread of the tv's. In this research, the tv was referred to as the length of time needed for mycelium to be visible, or the amount of time it took for the colonies to expand to a diameter of around 2 mm. While a criterion of 3 mm has been employed in certain research, other writers have also utilized this threshold. It is important to note that the tv's used in this study may have underestimated the actual amount of time needed for fruit goods to be rejected. The presence of additional fruit components, including organic acids and preservatives, reduced oxygen concentration, and other stress factors, which may possibly contribute to extended tv's, is not taken into consideration by the aPDA media. The tv values are also substantially less than the average shelf life of fruit goods, which are few months when stored at room temperature. The ascospores used in this study were subjected to a heat treatment involving a 10‐min heating shock at 80°C, which can be considered a form of pasteurization. While these pasteurization methods are not highly intense, they may still cause sub‐lethal damage to the ascospores, possibly resulting in prolonged periods for apparent development. Moreover, the tvs of the large inoculums employed in this work (about 100 ascospores each plate) are mostly represented by ascospores with very fast germination and lag durations and may greatly differ from contamination by one or a few spores. Therefore, it is crucial to confirm such findings in actual fruit items. Nonetheless, the worst‐case situations in our data are represented, which could be extremely helpful for creating fail‐safe predictive (shelf‐life) models.

## 5 CONCLUSION

To maintain or enhance the microbiological safety of food products, it is essential to explore intrinsic and extrinsic environmental factors that influence the growth and development of microbial species. This study investigated the impact of various processing conditions on the time required to detect growth/no growth data for three distinct HRM species isolated from fruit products. Additionally, parametric statistical distributions were employed to quantify the intrinsic diversity of individual ascospores and their time to develop observable growth. The findings revealed significant variations in individual timeframes for the formation of visible mycelia, particularly within the growth/no growth zones. The data collected in this study have implications for evaluating microbiological risk and predicting risk to prevent the deterioration of fruit‐based products due to HRM. However, future research endeavors should expand the scope to include longer incubation times, as extended shelf‐lives are critical for the food industry and consumers alike. Moreover, addressing the risk of mycotoxins' production is imperative for ensuring food safety. Additionally, broader inclusion of HRM species in future studies would enhance the generalizability of the findings.

## AUTHOR CONTRIBUTIONS

**Mahabub Alam:** Conceptualization (equal); data curation (equal); formal analysis (equal); investigation (equal); methodology (equal); resources (equal); software (equal); validation (equal); visualization (equal); writing – original draft (equal); writing – review and editing (equal). **Animesh Sarkar:** Conceptualization (equal); funding acquisition (equal); methodology (equal); project administration (equal); resources (equal); software (equal); supervision (equal); validation (equal); visualization (equal); writing – original draft (equal); writing – review and editing (equal). **A. S. M. Sayem:** Funding acquisition (equal); methodology (equal); project administration (equal); resources (equal); software (equal); validation (equal); writing – review and editing (equal). **Rahul Biswas:** Conceptualization (equal); data curation (equal); formal analysis (equal); methodology (equal); software (equal); validation (equal); writing – review and editing (equal). **M. M. Mahdi Hasan:** Data curation (equal); formal analysis (equal); writing – review and editing (equal).

## FUNDING INFORMATION

The research work was funded by the Ministry of Science and Technology, Bangladesh (Grant serial: 447 EAS).

## CONFLICT OF INTEREST STATEMENT

The authors declare no conflicts of interest.

## ETHICS STATEMENT

This study does not involve any human or animal testing.

## Data Availability

Upon a reasonable request, data would be made available.
